# Changes in Cortisol but Not in Brain-Derived Neurotrophic Factor Modulate the Association Between Sleep Disturbances and Major Depression

**DOI:** 10.3389/fnbeh.2020.00044

**Published:** 2020-04-28

**Authors:** Giuliana Travassos Pires Santiago, Ana Cecília de Menezes Galvão, Raíssa Nóbrega de Almeida, Sergio Arthuro Mota-Rolim, Fernanda Palhano-Fontes, João Paulo Maia-de-Oliveira, Dráulio Barros de Araújo, Bruno Lobão-Soares, Nicole Leite Galvão-Coelho

**Affiliations:** ^1^Laboratory of Hormonal Measurement, Department of Physiology and Behavior, Federal University of Rio Grande do Norte, Natal, Brazil; ^2^Postgraduate Program in Psychobiology, Federal University of Rio Grande do Norte, Natal, Brazil; ^3^Brain Institute, Federal University of Rio Grande do Norte, Natal, Brazil; ^4^Onofre Lopes University Hospital, Federal University of Rio Grande do Norte, Natal, Brazil; ^5^National Science and Technology Institute for Translational Medicine (INCT-TM), Natal, Brazil; ^6^Department of Clinical Medicine, Federal University of Rio Grande do Norte, Natal, Brazil; ^7^Department of Biophysics and Pharmacology, Federal University of Rio Grande do Norte, Natal, Brazil

**Keywords:** pittsburgh sleep quality index, brain-derived neurotrophic factor, salivary cortisol awakening response, sleep disturbance, treatment-resistant depression

## Abstract

Sleep disturbance is a symptom consistently found in major depression and is associated with a longer course of illness, reduced response to treatment, increased risk of relapse and recurrence. Chronic insomnia has been associated with changes in cortisol and serum brain-derived neurotrophic factor (BDNF) levels, which in turn are also changed in major depression. Here, we evaluated the relationship between sleep quality, salivary cortisol awakening response (CAR), and serum BDNF levels in patients with sleep disturbance and treatment-resistant major depression (*n* = 18), and in a control group of healthy subjects with good (*n* = 21) and poor (*n* = 18) sleep quality. We observed that the patients had the lowest CAR and sleep duration of all three groups and a higher latency to sleep than the healthy volunteers with a good sleep profile. Besides, low CAR was correlated with more severe depressive symptoms and worse sleep quality. There was no difference in serum BDNF levels between groups with distinct sleep quality. Taken together, our results showed a relationship between changes in CAR and in sleep quality in patients with treatment-resistant depression, which were correlated with the severity of disease, suggesting that cortisol could be a physiological link between sleep disturbance and major depression.

## Introduction

Changes in quality, duration, and structure of sleep are frequently observed in patients with mood disorders. In major depression, sleep alterations are part of the diagnosis, and are associated with depressed mood and loss of interest or pleasure (Sadock et al., 2016). Studies have pointed out that sleep problems usually precede other symptoms of depression and that the quality of sleep worsens before the onset of the first depressive episode (Breslau et al., 1996; Perlis et al., 1997; Ohayon and Roth, 2003). Moreover, during a depressive episode, persistent sleep disturbances are associated with more severe symptoms, worse treatment outcomes, increased risk of relapse and recurrence, and longer course of illness (Moos and Cronkite, 1999).

Among different sleep disorders, insomnia is the most frequent in patients with major depression (Harvey, 2001; Lucchesi et al., 2005). Normally, the diagnosis of insomnia is based on the difficulty to initiate or maintain sleep, which in turn results in reduced duration and quality of sleep (Schutte-Rodin et al., 2008). Some patients with depression do not present a clinical diagnosis of insomnia but show frequent nocturnal arousals, reduction in total sleep duration, non-restorative sleep, or disruptive dreams, often associated with suicidal ideation (Agargun et al., 2003; Chellappa and Araujo, 2007b). On the other hand, about 10–20% of patients with depression present hypersomnia, which is characterized by increases in night sleep duration and excessive daytime sleepiness (Pelayo, 2014).

Specific neurotransmitters and hormones, such as serotonin (Ursin, 2002), histamine and adenosine (Huang et al., 2007), cortisol (Weitzman et al., 1971; Steiger, 2003), and melatonin (Weinberg et al., 1979; Dubocovich, 2007) are associated with the modulation of sleep. The hypothalamus-pituitary-adrenal (HPA) axis plays an important role in the sleep–wake cycle. Cortisol, the final hormone of the HPA axis, presents a circadian rhythm with maximal secretion in the morning, before arousal, and lower levels at the end of the day, which are important for sleep onset (Born and Fehm, 2000; Palma et al., 2007; Gotlieb et al., 2018).

HPA axis dysregulation is commonly associated with sleep disorders (Chrousos et al., 1993; Tsuchiyama et al., 1995). Chronic insomnia is associated with circadian cortisol changes and hypercortisolemia, mainly in the evening (Vargas et al., 2018). However, enough homeostatic cortisol level has an important role in the induction and maintenance of rapid eye movement (REM) sleep (García-Borreguero et al., 2000). Moreover, hypersecretion of the corticotrophin-releasing factor (CRF)—a hormone that starts HPA axis function—is also related to sleep changes (Held et al., 2004; Holsboer and Ising, 2008).

In major depression, large changes in the HPA axis are related with chronic stress, depression severity, and duration of illness (Bhagwagar et al., 2005; Bremmer et al., 2007; Mayer et al., 2018). Increased cortisol awakening response (CAR) and hypercortisolemia are the most often reported changes in the HPA axis of patients with major depression, suggesting hyperactivity of this axis. This change could be in part due to a weak feedback sensibility and consequently CRF hypersecretion (Bhagwagar et al., 2005; Gillespie and Nemeroff, 2005; Holsboer and Ising, 2008). However, some depressive patients also have blunted CAR and hypocortisolemia (Huber et al., 2006; Bremmer et al., 2007). Both cortisol and CRF are linked with monoaminergic systems, and their changes could be related to the reduction of monoamines in the synaptic cleft, the main theory of major depression physiopathology (Steckler et al., 1999; Jeon and Kim, 2016). Therefore, since homeostatic cortisol levels and its circadian oscillations seem to be essential to a good sleep function, cortisol changes have been studied as a common physiological marker between major depression and sleep disorders (Vargas et al., 2018).

Moreover, brain-derived neurotrophic factor (BDNF), a neurotrophin associated with neuroplasticity, has recently become recognized as an indicator of sleep quality (Giese et al., 2014). Serum BDNF levels are often reduced in sleep disorders, as insomnia (Fan et al., 2018; Mikoteit et al., 2019), probably due to increased stress, which in turn reduces neurotrophic factor levels (Giese et al., 2013). The homeostatic level of cortisol is necessary for appropriate BDNF function, but higher levels of cortisol during chronic stress biding to glucocorticoid receptors reduces BDNF expression (Hansson et al., 2000; Kino et al., 2010). However, *in vivo*, this biphasic regulation may be more complex and depends on the brain area: in the amygdala, for instance, high levels of cortisol induce neurogenesis (Adzic et al., 2009).

BDNF has been considered an important biomarker of major depression (Polyakova et al., 2015; Peng et al., 2018). Preclinical and clinical studies of depressive patients have found low levels of blood BDNF, mainly in non-treated patients, when compared with those of healthy volunteers, and changes in BDNF levels are related to treatment response (Kreinin et al., 2015).

Despite some studies suggesting that patients with major depression have alterations in cortisol and BDNF levels (Leuchter et al., 2010; Tadić et al., 2011; Herbert, 2013), there is no consensus (Pariante et al., 2004; Molendijk et al., 2011; Elfving et al., 2012; Papakostas et al., 2013). To help clarify this issue, here we evaluated the relationship between sleep quality and disturbances, salivary CAR, and serum BDNF levels in patients with treatment-resistant major depression, compared with healthy volunteers. We hypothesize that patients will present alterations in salivary CAR and serum BDNF, which will be positively correlated with depression severity and sleep disturbances.

## Materials and Methods

This study was approved by the Research Ethics Committee of the Onofre Lopes University Hospital (HUOL), Federal University of Rio Grande do Norte (UFRN), Natal, RN, Brazil (#579.479). All volunteers agreed to and signed the informed consent form prior to participation. The procedures of this study comply with the ethical standards of the relevant national and institutional committees for human experimentation and with the Declaration of Helsinki of 1975, revised in 2008.

All patients presented treatment-resistant major depression (MD group), defined here as at least two unsuccessful treatments with commercially available antidepressants from different classes. Patients (MD; 4 men and 14 women) were in a currently moderate-to-severe depressive episode during the study, according to the Hamilton Depression Scale (HAM-D; Hamilton, 1960) and presented sleep disturbances, according to the Pittsburgh Sleep Quality Index (PSQI).

Although the HPA axis and BDNF synthesis are not the main targets for antidepressants actions, they seem to modulate these two pathways (Schüle et al., 2006; Schüle, 2007; Wilkinson et al., 2018). Therefore, we required to all patients complete an average of 2-week washout period, adjusted to the half-life time of their current medications. Therefore patients were not taking antidepressants during this study. However, they were allowed to use benzodiazepines if experiencing withdrawal symptoms.

The control group of healthy volunteers (CG; 17 men and 22 women) had no previously diagnosed neurological or psychiatric disorders and did not present sleep disturbances according to the PSQI.

Patients and healthy individuals were matched by age and socio-demographic criteria. The following inclusion criteria were adopted for both groups: minimum of 18 years of age and absence of a present or past history of substance addiction. Exclusion criteria included the following: imminent suicidal risk, bipolar affective disorder, presence of psychotic symptoms, history of manic or hypomanic symptoms, and family history of schizophrenia.

### Experimental Design

After screening and washout, volunteers were admitted to the hospital research unit for one overnight stay, in which they slept in a room (D1) reserved and prepared for the study. At D1, the Montgomery–Åsberg Depression Scale (MADRS) was applied to all volunteers to assess depression severity (Rush et al., 2004; Howland, 2008; Palhano-Fontes et al., 2019). The PSQI and Epworth Sleepiness Scale (ESS) were applied at D1 before sleep onset. The duration of sleep and the presence of nocturnal awakening were recorded. On the following morning, at awakening around 6 AM, saliva was collected for CAR. Additionally, blood samples were provided for serum BDNF quantification, when all subjects were fasting for at least 8 h.

### Sleep Psychometric Instruments

The PSQI is a self-assessment scale that accesses sleep quality from the previous month. The questions measure seven clinical components: subjective sleep quality (C1), latency (C2), duration (C3), sleep efficiency (C4), sleep alterations (C5), medication use (C6), and daytime sleep dysfunction (C7). Overall score ranges from 0 to 21 points, which is classified as good sleep quality (from 0 to 4 points), poor sleep quality (from 5 to 10 points), and sleep disturbance (if above 10 points). Besides total PSQI score, we analyzed sleep latency and duration of sleep, measured by the PSQI.

The ESS was applied to evaluate daytime sleepiness. ESS consists of eight statements rated from 0 to 3. A null score implies no chances of taking a nap, whereas scores of 1, 2, and 3 mean small, moderate and high chance of a nap throughout the day, respectively.

### Salivary Cortisol Awakening Response

Saliva was used to analyze the CAR. It was obtained through a Salivette (Sarstedt AG & Co., KG Werk Hemer, Germany), a device specifically used for saliva collection composed of a cotton swab and a plastic tube. Three samples of saliva were collected: immediately at awakening, 30 and 45 min after awakening. Volunteers were not allowed to move excessively, brush their teeth, eat, or drink before or during saliva collection. Saliva samples were centrifuged (15 min, 4°C to 3,000 rpm) and stored in aliquots of 0.300 ml, at −80°C. Salivary cortisol was measured with competitive ELISA kit DGR-SLV 4635.

### Serum Brain-Derived Neurotrophic Factor

Blood collection was performed with a disposable syringe by trained professionals from the HUOL clinical analysis laboratory. Blood samples were centrifuged (10 min, 4°C to 3,000 rpm), and the serum samples were stored in aliquots of 0.300 ml at −80°C. BDNF quantification was performed by sandwich ELISA with the Merck Millipore CYT306 kit.

### Statistical Analysis

For CAR statistical analysis, the area under the curve (AUC) was calculated in cm^3^, using the three cortisol concentration measurements. Quantitative dependent variables investigated in this study are as follows: total PSQI score and its seven components, previous month sleep latency and sleep duration measured by the PSQI, daytime sleepiness, sleep duration at D1, CAR, and serum BDNF levels. PSQI, previous month sleep duration, sleep duration at D1, CAR, and serum BDNF levels were normalized by logarithmic transformation. The nocturnal awakening at D1 (present or not) was treated as a categorical dependent variable.

All volunteers (CG and MD) were categorized with respect to sleep quality, according to the PSQI total score, into three groups: good sleep (GS), poor sleep (PS), and sleep disturbance (SD). To analyze the influence of previous antidepressants treatments, patients were split into three groups, which were included as categorical independent variables: the number of unsuccessful previous antidepressant treatments with different drug classes, G1 (two or three previous treatments, *n* = 10), G2 (four or five previous treatments, *n* = 5), and G3 (six to nine previous treatments, *n* = 3). These groups and gender were included as categorical independent variables.

The influence of gender and sleep quality (GS, PS, and SD) on PSQI scores and CAR was explored with a general linear model (GLM) and Fisher *post hoc* test. For BDNF, we applied the analysis of covariance (ANCOVA) using groups (GS, PS, and SD) and gender as independent variables and the levels of platelets as covariate. A one-way analysis of variance (one-way ANOVA) and a Fisher *post hoc* test were used to investigate possible differences in sleep duration according to sleep quality (GS, PS, and SD) and to CAR and BDNF levels with respect to the number of unsuccessful previous treatments.

A Student’s *t*-test was used to investigate possible differences in PSQI, CAR, and BDNF with respect to comorbid personality and anxiety disorders. To analyze daytime sleepiness and previous month sleep latency with respect to group sleep quality (GS, PS, and SD), we applied the Kruskal–Wallis test. The chi-square test was used to cross the nocturnal awakenings at D1 with respect to sleep quality (GS, PS, and SD). Multiple regression was applied to analyze the modulation of the patients’ previous benzodiazepines treatment (yes or no) on CAR and BDNF levels.

Spearman’s correlation was applied to analyze possible correlations between PSQI, and its seven components, previous month sleep latency and sleep duration, daytime sleepiness, sleep duration at D1, CAR and serum BDNF levels, and them with MADRS scores, considering the groups together (GS, PS, and SD).

Statistical analysis was conducted in SPSS 20. Graphics were built in R 3.4.1 (RStudio). The level of significance was set at *p* ≤ 0.05 (two-tailed).

## Results

All volunteers were Brazilians adults (MD, 39.83 ± 11.6 years old; CG, 31.6 ± 9.6 years old), and the majority were women (MD, 78%; CG, 56.4%). Compared with controls, who had higher education (MD, 16.67%; CG, 33.33%), patients had predominantly secondary education (MD, 61.11%; CG, 17.94%) and lower income (MD, 83.33%; CG, 51.28%).

On average, patients presented 8.11 ± 7.10 years of depression, with 13.94 ± 17.07 previous episodes and an average MADRS score of 33.27 ± 6.14 at the time of the study. Patients had been previously treated on average with 4.05 ± 1.98 different types of antidepressant medications.

All patients (100%) had been treated already with a selective serotonin reuptake inhibitor (SSRI), 50% also used tricyclic antidepressants (TCAs), 44.44% used serotonin–norepinephrine reuptake inhibitor (SNRI), and 16.67% had tried norepinephrine–dopamine reuptake inhibitor (NDRI). The percentage of antidepressants used does not add up to 100% owing to the overlapping with previous antidepressant treatments. All patients were using benzodiazepines hypnotics/sedatives (alprazolam, *n* = 2; bromazepam, *n* = 2; and diazepam, *n* = 2) and antiepileptic (clonazepam, *n* = 12). Nine patients were already treated with benzodiazepines before this study and nine were not.

### Pittsburgh Sleep Quality Index

**Figure 1:**
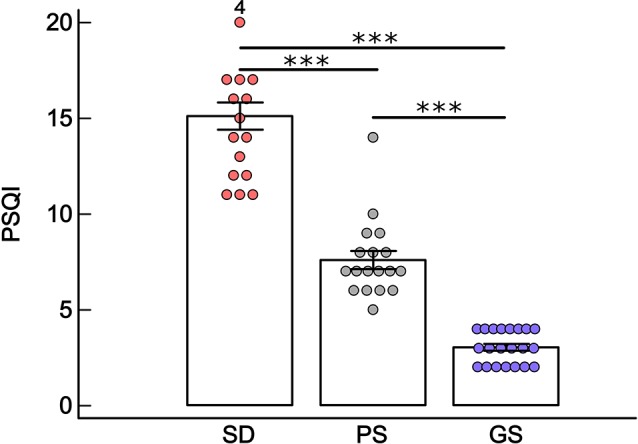
Mean ± standard error of Pittsburgh Sleep Quality Index (PSQI) in the sleep disturbance (SD: *n* = 18 depressive patients), poor sleep (PS: *n* = 18 healthy volunteers) and good sleep (GS: *n* = 21 healthy volunteers) groups. ***p < 0.001, general linear model (GLM) and Fisher *post hoc*. The number 4 on first bar indicates volunteers that have score higher than 20.

All MD patients showed sleep disturbances. In the control group, 18 individuals had poor sleep quality, while 21 showed good sleep. With respect to gender, 4 men and 14 women showed sleep disturbance; 7 men and 11 women presented poor sleep and 10 men and 11 women had good sleep. The sleep disturbance group presented higher total PSQI scores than did the poor sleep and good sleep groups (GLM; Group*: *F* = 122.67, *df* = 2, *p* < 0.001; Fisher *post hoc*, SD*PS, *p* < 0.001; SD*GS, *p* < 0.001); and the PS group presented higher PSQI scores than the GS (Fisher *post hoc*, *p* < 0.001; Figure 1). There were no significant differences observed in PSQI scores with respect to gender and to the interaction between group and gender. For all statistical values of main effects and interactions of GLM and for descriptive statistic, see Supplementary Tables S1, S2.

Three patients had comorbid diabetes mellitus, but all showed controlled levels of glycemia (according to reference values) during the study, analyzed by fast glucose levels. Half of the patients had comorbid anxiety disorder (*n* = 9) and 77% had a comorbid personality disorder (13 patients with personality disorder in cluster B and one patient in cluster A). No significant differences were found in PSQI with respect to comorbid anxiety disorder (Student’s *t*-test: *t* = −0.94, *df* = 16, *p* = 0.35) and comorbid personality disorder in cluster B (Student’s *t*-test: *t* = 0.67, *df* = 15, *p* = 0.50; Supplementary Table S3).

**Figure 2:**
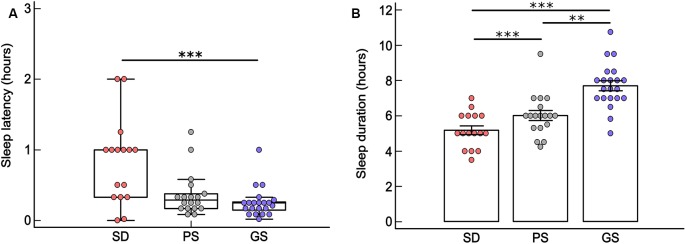
**(A)** Box plot (median ± Q75% and Q25%) of last month sleep latency (in hours) according to the Pittsburgh Sleep Quality Index (PSQI) in the sleep disturbance (SD: *n* = 16), poor sleep (PS: *n* = 18) and good sleep (GS: *n* = 21) groups. ***p < 0.001, Kruskal–Wallis test. **(B)** Mean ± standard error of the previous month sleep duration (in hours) according to the PSQI, in SD (*n* = 16) PS (*n* = 18), and GS (*n* = 21) groups. ***p < 0.001 and **p < 0.05 one-way analysis of variance (ANOVA) and Fisher *post hoc*. Two outlier patients of SD were excluded from both statistical analyses.

The SD group presented higher previous month sleep latency than did the GS group (Kruskal–Wallis; *H* = 12.67, *df* = 2, *p* < 0.001; SD*GS, *p* < 0.001). A trend toward significance was observed in latency to sleep between the SD and PS groups (*p* = 0.08) but no significant difference between the GS and PS groups (*p* = 0.56; Figure 2A, Supplementary Table S2). Two outlier patients in the SD group who showed 3 and 4 h of sleep latency were excluded from the statistical analysis.

The GS group showed longer sleep duration during the previous month than the SD and PS groups (one-way ANOVA: *F* = 22.02, *df* = 2, *p* < 0.001; Fisher *post hoc*, GS*PS: *p* < 0.05; GS*SD, *p* < 0.001). The PS group showed longer sleep duration during the previous month than did the SD group (Fisher *post hoc*; PS*SD, *p* < 0.001). Two outlier patients in the SD group who showed 10 and 12 h of sleep duration were excluded from the statistical analysis (Figure 2B, Supplementary Table S2).

Considering all groups together (SD, PS, and GS), significantly positive correlations were found between MADRS scores, and a) PSQI total score (*Spearman* rho = 0.72, *p* ≤ 0.05) and b) previous month sleep latency (Spearman rho = 0.52, *p* ≤ 0.05). Moreover, significant negative correlations were found between MADRS scores and previous month sleep duration (Spearman rho = −0.31, *p* ≤ 0.05). All results of statistical Spearman correlation are in Table 1.

**Table 1:** Statistical values of Spearman correlation tests between quantitative dependent variables considering all groups together [good sleep (GS), poor sleep (PS) and sleep disturbance (SD)].

	CAR	BDNF	PSQI	Latency	Duration	Sleepiness	Duration D1
MADRS	−0.520*	0.060	0.720*	0.520*	−0.310*	0.009	−0.020
CAR	-	−0.060	−0.340*	0.220	−0.240	0.170	0.090
BDNF	−0.60	-	0.020	−0.020	−0.150	0.080	0.060
PSQI	−0.340*	0.020	-	-	-	0.160	−0.080
Latency	−0.240	−0.020	-	-	-	−0.070	0.100
Duration	0.220	−0.150	-	-	-	−0.170	0.130
Sleepiness	0.170	0.080	0.160	−0.070	−0.170	-	0.140

*Molecular biomarkers: blood brain-derived neurotrophic factor (BDNF) and salivary cortisol awakening response (CAR). Depressive symptoms: Montgomery–Åsberg Depression Scale (MADRS). Sleep quality: total Pittsburgh Sleep Quality Index (PSQI), latency to sleep (from PSQI), sleep duration (from PSQI and at D1), and daytime sleepiness. D1: hospital overnight; groups: sleep disturbance (SD, *n* = 18), poor sleep (PS, *n* = 18), and good sleep (GS, *n* = 21). Bold text indicates significance: *p* ≤ 0.05*.

### Daytime Sleepiness (by Epworth Sleepiness Scale)

**Figure 3:**
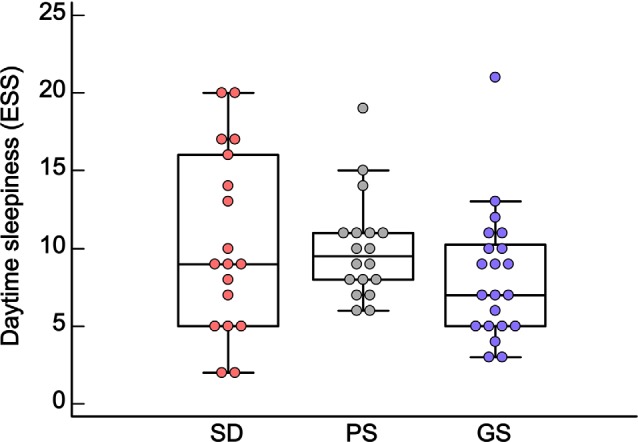
Box plot of daytime sleepiness (median ± Q75% and Q25%), according to the Epworth Sleepiness Scale (ESS), in the sleep disturbance (SD: *n* = 18), poor sleep (PS: *n* = 18) and good sleep (GS: *n* = 21) groups. *p* ≥ 0.05, Kruskal–Wallis test.

No statistically significant differences were observed for daytime sleepiness between groups (Kruskal–Wallis; *H* = 2.80, *df* = 2, *p* = 0.24; Figure 3 and Supplementary Table S2).

Considering all groups together (SD, PS, and GS), no significant correlations were found between scores of daytime sleepiness and the other quantitative dependent variables: MADRS, CAR, BDNF, PSQI, sleep latency, and sleep duration (Spearman *p* ≥ 0.05; Table 1).

### Sleep Duration at D1

**Figure 4:**
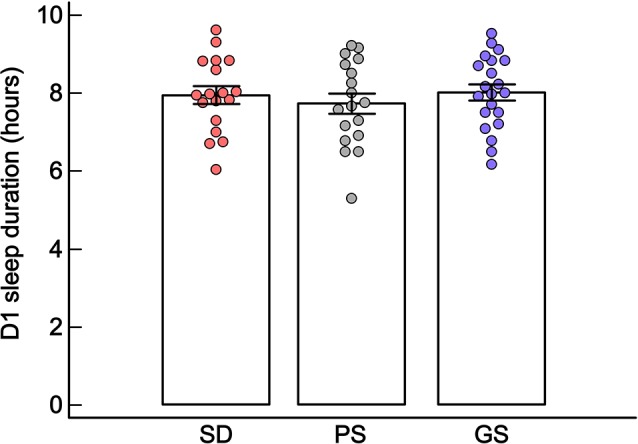
Mean ± standard error of sleep duration (in hours) at D1, in the sleep disturbance (SD: *n* = 18), poor sleep (PS: *n* = 18) and good sleep (GS: *n* = 21) groups. *p* ≥ 0.05, one-way ANOVA.

No statistically significant differences were observed regarding sleep duration at D1 between SD, PS, and GS (one-way ANOVA; *F* = 0.42, *df* = 2, *p* = 0.65; Figure 4 and Supplementary Table S2).

Considering all groups together (SD, PS, and GS), no significant correlations were found between sleep duration at D1 and the other quantitative dependent variables: MADRS, CAR, BDNF, PSQI, sleep latency, and sleep duration (Spearman *p* ≥ 0.05; Table 1).

### Nocturnal Awakening at D1

Sleep quality at D1 showed that nocturnal awakening was present in 50% of the individuals with sleep disturbance (*n* = 9), in 42.85% (*n* = 9) of the individuals with poor sleep quality, and in 38.8% (*n* = 7) of the individuals with good sleep quality. No statistically significant differences were observed for nocturnal awakening between groups (chi-square: *χ*^2^ = 6.36, *p* = 1).

### Salivary Cortisol Awakening Response

**Figure 5:**
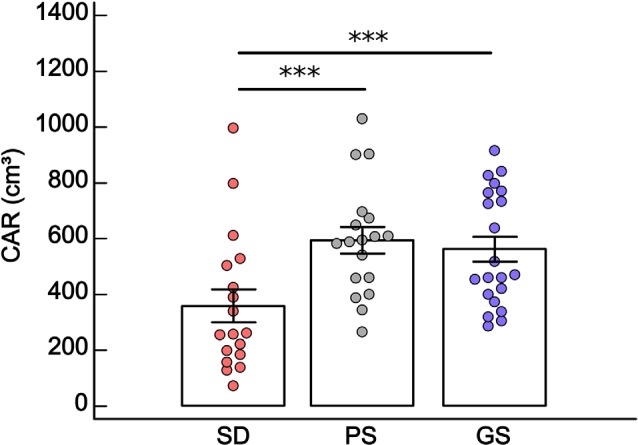
Mean ± standard error of salivary cortisol awakening response (CAR: cm^3^) in the sleep disturbance (SD: *n* = 18), poor sleep (PS: *n* = 18) and good sleep (GS: *n* = 21) groups. ***p < 0.001, general linear model (GLM) and Fisher *post hoc*. Two patients in SD showed close values, one of them being superimposed on the graph.

Volunteers with sleep disturbance had lower salivary CAR than subjects with poor sleep (*F* = 7.31, *df* = 2, *p* < 0.001, Fisher *post hoc*, *p* < 0.001) and good sleep quality (Fisher *post hoc*, *p* < 0.001). There was no difference in CAR between PS and GS (Fisher *post hoc*, *p* = 0.69). No differences in CAR were observed with respect to gender and for the interaction between group and gender (Figure 5). For all statistical values of main effects and interactions of GLM and for descriptive statistic, see Supplementary Tables S1, S2.

No differences were observed in CAR with respect to the number of previous antidepressant treatments (one-way ANOVA: *F* = 0.28, *df* = 2, *p* = 0.75; G1: *n* = 10, *μ* = 354.4 ± 80.9 cm^3^; G2: *n* = 5, *μ* = 296.2 ± 114.5 cm^3^; G3: *n* = 3, *μ* = 479.4 ± 147.8 cm^3^; Supplementary Figure S1). Moreover, previous treatment with benzodiazepines did not predict CAR (multiple regression: AICC = 198.86, *p* = 0.08).

No difference was found in CAR between patients with and without comorbid anxiety disorder (*t*-test: *t* = −0.12, *df* = 16, *p* = 0.90) and comorbid personality disorder (*t*-test: *t* = −0.82, *df* = 15, *p* = 0.42; Supplementary Figure S2, Supplementary Table S3).

Considering all groups together (GS, PS, and SD), there were significant negative correlations between CAR and MADRS scores (Spearman rho = −0.52, *p* ≤ 0.05) and PSQI total score (Spearman rho = −0.34, *p* ≤ 0.05; Table 1). Moreover, negative significant correlations were observed between CAR and some clinical components of the PSQI; subjective sleep quality (C1), sleep efficiency (C4), sleep alterations (C5), and medication use (C6; Supplementary Table S4). Higher scores in these components mean worse sleep quality.

### Serum Brain-Derived Neurotrophic Factor Levels

**Figure 6:**
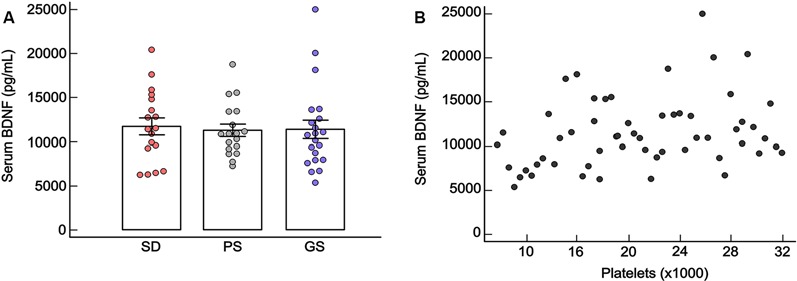
**(A)** Mean ± standard error of serum blood brain-derived neurotrophic factor (BDNF) levels (pg/ml) in the sleep disturbance (SD: *n* = 18), poor sleep (PS: *n* = 18) and good sleep (GS: *n* = 21). *p* ≥ 0.05, ANCOVA. **(B)** Statistically significant Spearman correlation between serum BDNF levels and platelets (rho = 0.29, *p* ≤ 0.05).

There were no significant differences in BDNF levels between the SD, PS, and GS groups (ANCOVA: *F* = 0.19, *df* = 2, *p* = 0.82). Platelets were statistically significant as covariable (ANCOVA: *F* = 6.49, *df* = 1, *p* = 0.01), and a positive correlation for serum BDNF levels and platelets was observed (Spearman rho = 0.29 *p* ≤ 0.05). No significant differences were observed in BDNF levels with respect to gender nor for the interaction between group and gender (Figure 6). For all statistical values of main effects and interactions of GLM and for descriptive statistic, see Supplementary Tables S1, S2.

No significant differences were found for BDNF levels with respect to previous antidepressant treatments (one-way ANOVA: *F* = 2.63, *df* = 2, *p* = 0.10; G1: *n* = 10, *μ* = 13,355.23 ± 1,967.26 pg/ml; G2: *n* = 5, *μ* = 11,222.27 ± 2,782.12 pg/ml; G3: *n* = 3, *μ* = 8,648.96 ± 3,591.71 pg/ml; Supplementary Figure S3). Additionally, previous treatments with benzodiazepines did not predict BDNF (multiple regression: AICC = 198.86, *p* = 0.08).

No differences were observed for BDNF levels between patients with and without comorbid anxiety disorder (*t*-test: *t* = −1.13, *df* = 16, *p* = 0.27) and comorbid personality disorder (*t*-test: *t* = 2.09, *df* = 15, *p* = 0.055; Supplementary Figure S4, Supplementary Table S3).

Considering all groups together (GS, PS, and SD), there were no statistically significant correlations to BDNF and CAR, MADRS, or dependent variables of sleep quality (Table 1, Supplementary Table S4).

## Discussion

In this study, we observed that all patients with major depression had sleep disturbances, whereas 54% of the healthy controls had poor sleep quality. We found that patients had shorter time of total sleep and higher sleep latency than control volunteers with good sleep quality, which were associated with more severe symptoms of depression. Moreover, patients showed lower salivary CAR than did healthy controls, which was correlated with more severe depressive symptoms and worse sleep quality. Comorbid anxiety and personality disorders did not modulate the sleep quality of the patients, CAR, or BDNF levels. Moreover, the number of unsuccessful previous antidepressant treatments with different drug classes and previous treatments with benzodiazepines were not related with CAR and BDNF levels.

The higher number of women than men in our sample reflects the prevalence of major depression in the general population (Panagiotakopoulos and Neigh, 2014; Kuehner, 2017). However, gender did not modulate salivary CAR nor serum BDNF levels in our sample, although sex hormones have been shown to modulate both BDNF transcription and cortisol secretion (Kudielka and Kirschbaum, 2005; Atwi et al., 2014; Kreinin et al., 2015; Glud et al., 2019). Therefore, the link between these biomarkers, sleep disturbance and major depression, with respect to gender, requires further evaluation (Kreinin et al., 2015; Galvão et al., 2018; Almeida et al., 2019).

Our results show the presence of insomnia in the patients, particularly of initial insomnia, as our patients had higher latency to sleep than healthy controls with good sleep, and the lowest sleep duration with respect to controls with good and poor sleep. These results corroborate the literature that suggests the link between sleep disturbances, mainly insomnia, and major depression (Thase, 1999; Taylor et al., 2005; Alvaro et al., 2013). A previous study assessed 70 patients with major depression (44 women and 26 men) and found out that 70% of them had insomnia, with a significant positive correlation between PSQI scores and depression severity (Chellappa and Araujo, 2007a). Insomnia complaints are frequent in major depression (Taylor et al., 2005; Alvaro et al., 2013; Gebara et al., 2018), especially initial insomnia, which has been correlated with anxiety and personality traits (Chellappa and Araujo, 2007a; Harvey et al., 2014; Van Veen et al., 2017). In fact, most of our patients had comorbid personality disorder and/or anxiety disorder. However, none of these modulated PSQI.

Herein, we did not observe significant differences in daytime sleepiness when comparing patients with sleep disturbance and healthy controls with poor or good sleep quality. Moreover, sleep quality at the night spent in the hospital show that patients with sleep disturbances and healthy controls had similar nocturnal awakenings and sleep duration. Patients’ mean sleep duration was 2 h longer than recorded in the previous month by the PSQI, whereas healthy subjects showed approximately 40 min longer sleep duration. Since most volunteers had low socioeconomic status, the room conditions offered were probably more comfortable than what they had at their homes, which may have influenced positively their sleep duration and quality.

The relationship between sleep disturbances and major depression is complex, mainly in treatment-resistant patients, as is the case of our sample; however, usually, sleep alterations are a core symptom of the disease than a side effect of antidepressant medication (Kurian et al., 2010). Patients with residual symptoms of sleep disorders, as insomnia, have higher chances of recurrence than those without persistent sleep disturbances (Judd et al., 1998; Kurian et al., 2010). Likewise, the absence of response to pharmacological treatment and recurrence are related to chronic sleep disturbances (Fawcett et al., 1990; Buysse et al., 1997, 1999). It is important to highlight that the literature appoints to a good validity of the PSQI when compared with more objective measurements such as polysomnography, which is considered the gold standard in the field (Mollayeva et al., 2016). However, in some cases, subjective changes in sleep have been present even without changes measured by polysomnography (Argyropoulos et al., 2003). Therefore, our findings corroborate the relevance of using subjective sleep evaluation scales, such as the PSQI, as a clinical tool for sleep investigation in major depression, because polysomnography analyses require a sophisticated laboratory setup as well as subjects’ availability to sleep in the study setting.

The sleep–wake cycle is regulated by the interaction between homeostatic and circadian processes (Borb and Achermann, 1999), and changes in these mechanisms can induce sleep-related complaints and disorders. The suprachiasmatic nucleus of the hypothalamus regulates the oscillating pattern of cortisol release, with high levels before awakening and low levels at night. Disturbances in circadian rhythms of cortisol levels can change the 24-h sleep–wake cycle, which in turn has been related to the etiology of mood disorders (Luca et al., 2013). We found lower salivary CAR in the group of patients with sleep disturbances, than in healthy subjects with good and bad sleep quality. No difference was observed in CAR levels between healthy controls with good and bad sleep. Moreover, lower CAR was positively correlated with depression severity and worse sleep quality, inferred by total PSQI score and some of its components such as subjective sleep quality, sleep efficiency, sleep alterations, and medication use.

Some studies showed that sleep disturbances, like insomnia, are correlated with reduced CAR in subjects without psychiatry disorders (Backhaus et al., 2004). Hypocortisolemic addisonian patients treated with hydrocortisone reverted the sleep abnormalities, suggesting that homeostatic cortisol levels have permissive action on sleep (Gan and Pearce, 2016). Despite these associations, it is uncertain whether sleep disturbances alter cortisol levels or vice versa. However, we observed that sleep duration and nocturnal awakenings at D1 did not modulate salivary CAR on the next morning. Studies relating CAR to specific characteristics of the previous night have been very inconsistent (Elder et al., 2016). Some report that acute changes in sleep do not immediately modulate CAR, which tends to follow a more stable pattern of response (Dettenborn et al., 2007; Fries et al., 2009).

Disrupted HPA axis, advancement or delay in circadian rhythm of cortisol, and disturbance in upper and lower bounds of cortisol range are also observed in major depression (Abell et al., 2016; Starr et al., 2017). In some cases, depression is accompanied by hypocortisolemia (Bremmer et al., 2007; Vreeburg et al., 2013; Moreira et al., 2016; Galvão et al., 2018), which may be induced by prolonged use of particular type of antidepressants, such as mirtazapine—a selective antagonism at 5-HT_2_ receptors (Schüle et al., 2006). In other cases, prolonged exposure to stressors can induce high and chronic reactivity of the HPA axis, followed by adrenal exhaustion (Pariante et al., 2004; Wu et al., 2008; Leuchter et al., 2010). However, as our patients were under washout to antidepressants and the number of unsuccessful previous antidepressant treatments with different drug classes did not modulate CAR, we propose that hypocortisolemia found here is probably a symptom of the disease rather than a result of the antidepressant medication. However, it has been suggested that cortisol changes are a state of major depression, not a trait, owing to its dependence on disease’s phase, duration, and severity (Gillespie and Nemeroff, 2005).

Moreover, it is important to highlight that all patients were under benzodiazepines during this study and that half of them were already using them before the study. However, previous treatment with benzodiazepines did not predict CAR, corroborating some similar studies that did not observe changes in CAR induced by benzodiazepines (McIntyre et al., 1993; Manthey et al., 2010). Benzodiazepine modulation on the HPA axis is conflicting and dependent on the type of drug, dose, type of biological sample, and study design (Laakmann et al., 1984; Kalogeras et al., 1990; Pomara et al., 2005; Fries et al., 2006).

Nevertheless, as the normalization of the HPA function seems to be a prerequisite for the remission of depression, and it is important to sleep homeostasis, new treatments with focus in glucocorticoid stabilization should be stimulated (Steckler et al., 1999; Galvão et al., 2018). Depressive patients treated with antagonist drugs of the CRF receptor showed improvement in depressive symptoms and sleep disorders (Held et al., 2004; Holsboer and Ising, 2008).

Contrary to our hypothesis, there was no statistically significant difference in serum BDNF levels between groups with distinct sleep quality. Although not unanimous, studies have been consistently reporting low serum BDNF in subjects with chronic insomnia, which is correlated to poor subjective sleep quality and decreased REM sleep (Giese et al., 2013, 2014; Klein et al., 2013; Rethorst et al., 2015; Mikoteit et al., 2019). However, it has also been suggested that BDNF is not directly correlated to a specific sleep disorder, or sleep duration, but with the proportion of sleep stages and REM sleep (Deuschle et al., 2018). Therefore, our result might be partially contaminated by the short washout period and long-term use of antidepressants (Dubovsky, 2018; Wilkinson et al., 2018). The number of unsuccessful previous antidepressant treatments with different drug classes did not modulate BDNF levels. Furthermore, previous treatment with benzodiazepines did not predict BDNF, and in some cases, benzodiazepines are associated with memory impairment and reduction of BDNF expression in the hippocampus; thus, further studies should analyze this topic (Zafra et al., 1991; Licata et al., 2013).

We found a positive significant correlation between serum BDNF and platelets, and it is known that in the periphery, BDNF is mainly produced by platelets (Nurden et al., 2008). Nevertheless, we did not observe correlations between CAR and serum BDNF, although the literature appoints a biphasic model for the interaction between the HPA system and BDNF synthesis, besides the importance of cortisol to BDNF receptor activation (Kunugi et al., 2010; Giese et al., 2013; Schmitt et al., 2016).

It is essential to point out some limitations of this study. First, with respect to the sample size, our study was carried out with a modest number of patients, with treatment-resistant depression and some cases of comorbid personality and anxiety disorder. Although patients were under washout to antidepressants, they were under treatment with benzodiazepines. With respect to the study design, sleep was not assessed with polysomnography. Only one biological sample per volunteer was collected and pro-BDNF and mature BDNF molecules, which are more specific than total BDNF, were not dosed (Angoa-Pérez et al., 2017). Thus, further studies that address these limitations are necessary to obtain potentially relevant additional results.

## Conclusion

Our results suggest a relationship between changes in CAR and sleep quality in patients with treatment-resistant major depression, and changes in both were correlated with the severity of depression, suggesting that cortisol could be a potential physiological link between sleep disturbance and major depression.

## Data Availability Statement

The datasets generated for this study are available on request to the corresponding author.

## Ethics Statement

The studies involving human participants were reviewed and approved by the Research Ethics Committee of the Onofre Lopes University Hospital (HUOL), Federal University of Rio Grande do Norte (UFRN), Natal-RN, Brazil (#579.479), and was registered at http://clinicaltrials.gov (NCT02914769). The patients/participants provided their written informed consent to participate in this study.

## Author Contributions

NG-C, BL-S, DA, JM, and FP-F designed the experiments. RA and AC measured the hormonal data. FP-F, AG, and GS collected the experimental data, carried out the statistical analysis, and prepared the figures. NG-C, BL-S, DA, SM-R, FP-F, AM, and GS prepared the manuscript.

## Conflict of Interest

The authors declare that the research was conducted in the absence of any commercial or financial relationships that could be construed as a potential conflict of interest.

## Abbreviations

AUC: area under the curve
CAR: salivary cortisol awakening response
C1: subjective sleep quality
C2: sleep latency
C3: sleep duration
C4: sleep efficiency
C5: sleep alterations
C6: medication use
C7: daytime sleep dysfunction
D1: day which the volunteers slept
ESS: Epworth Sleepiness Scale
GC: control group
GS: volunteers with good sleep in PSQI
HUOL: Onofre Lopes University Hospital
MD: patients with treatment-resistant major depression
NDRI: norepinephrine–dopamine reuptake inhibitor
PS: volunteers with poor sleep in PSQI
PSQI: Pittsburgh Sleep Quality Index
REM: rapid eye movement
SD: volunteers with sleep disturbance in PSQI
SNRI: serotonin–norepinephrine reuptake inhibitor
SSRI: selective serotonin reuptake inhibitor
TCAs: tricyclic antidepressants
UFRN: Federal University of Rio Grande do Norte.
