# Supplementary material for: Changes in Cortisol but Not in Brain-Derived Neurotrophic Factor Modulate the Association Between Sleep Disturbances and Major Depression
Source: Front Behav Neurosci. 2020 Apr 28;14:44. doi: 10.3389/fnbeh.2020.00044 (PMC7199815; doi:10.3389/fnbeh.2020.00044)
Supplement: Supplementary file 1 [file Table_1.docx]

Supplementary table 1. Statistical values of main effects and interaction of multivariate analysis tested for Pittsburgh Sleep Quality Index (PSQI), salivary cortisol awakening response (CAR) and Brain-Derived Neurotrophic Factor (BDNF) between gender, for groups with good sleep (GS), poor sleep (PS) and sleep disturbance (SD).

| **EFFECT** | **F** | **p** | **DF** |
| --- | --- | --- | --- |
| **PSQI ^(GLM)^** |  |  |  |
| **Intercept** | **784.05** | **0.001** | **1** |
| **Group** | **122.67** | **0.001** | **2** |
| **Gender** | 0.015 | 0.90 | 1 |
| **Group*Gender** | 0.76 | 0.46 | 2 |
| **Error** |  |  | 51 |
| **CAR ^(GLM)^** |  |  |  |
| **Intercept** | **7131.94** | **0.001** | **1** |
| **Group** | **7.31** | **0.001** | **2** |
| **Gender** | 0.21 | 0.64 | 1 |
| **Group*Gender** | **0.11** | **0.89** | **2** |
| **Error** |  |  | 51 |
| **BDNF ^(ANCOVA)^** |  |  |  |
| **Group** | 0.19 | 0.82 | 2 |
| **Platelets** | **6.49** | **0.01** | **1** |
| **Gender** | 0.26 | 0.61 | 1 |
| **Group*Gender** | 1.35 | 0.26 | 2 |

^General Linear Models (GLM), analysis of co-variance (ANCOVA). *Bold text indicates significant statistics values^
