# Supplementary material for: Changes in Cortisol but Not in Brain-Derived Neurotrophic Factor Modulate the Association Between Sleep Disturbances and Major Depression
Source: Front Behav Neurosci. 2020 Apr 28;14:44. doi: 10.3389/fnbeh.2020.00044 (PMC7199815; doi:10.3389/fnbeh.2020.00044)
Supplement: Supplementary file 2 [file Table_2.docx]

| Supplementary table 2. Descriptive statistic (sample size, means ± SEM or median, Q25% and Q75%) of sleep quality quantitative dependent variables (hours), salivary cortisol awakening response (CAR, cm³) and Brain-Derived Neurotrophic Factor (BDNF, pg/mL) levels for groups with good sleep (GS), poor sleep (PS) and sleep disturbance (SD). | | | |
| --- | --- | --- | --- |
|  | **SD** | **PS** | **GS** |
| PSQI | N = 18  μ = 15.11 ± 0.49 | N = 18  μ = 7.61 ± 0.49 | N = 21  μ = 3.04 ± 0.45 |
| Sleep latency | N = 16  median = 1  Q25% = 0.33  Q75% = 11 | N = 18  median = 0.29  Q25% = 0.16  Q75% = 0.37 | N = 21  median = 0.25  Q25% = 0.16  Q75% = 1.25 |
| Sleep duration | N = 16  μ = 5.18 ± 0.29 | N = 18  μ = 6.01 ± 0.27 | N = 21  μ = 7.70 ± 0.25 |
| Daytime sleepiness | N = 18  median = 7.95  Q25% = 7.30  Q75% = 8.81 | N = 18  median = 7.71  Q25% = 6.91  Q75% = 8.73 | N = 21  median = 8  Q25% = 7.50  Q75% = 8.83 |
| Sleep duration (D1) | N = 18  μ = 7.8 ± 0.79 | N = 18  μ = 7.9 ± 1.08 | N = 21  μ = 8.3 ± 0.71 |
| CAR | μ = 346.29 ± 63.22 | μ = 593.31 ± 53.91 | μ = 560.15 ± 48.72 |
| BDNF | μ = 11145.11 ± 1120.23 | μ = 11397.24 ± 955.33 | μ = 11507.06 ± 888.10 |

^PSQI: Pittsburgh Sleep Quality Index, D1: Hospital overnight^
