# Supplementary material for: Changes in Cortisol but Not in Brain-Derived Neurotrophic Factor Modulate the Association Between Sleep Disturbances and Major Depression
Source: Front Behav Neurosci. 2020 Apr 28;14:44. doi: 10.3389/fnbeh.2020.00044 (PMC7199815; doi:10.3389/fnbeh.2020.00044)
Supplement: Supplementary file 3 [file Table_3.docx]

| Supplementary table 3. Mean ± SEM of Pittsburgh Sleep Quality Index (PSQI), and Salivary cortisol awakening response (CAR, cm³) and Brain-Derived Neurotrophic Factor (BDNF, pg/mL) levels for patients with and without comorbid anxiety disorder and comorbid personality disorder. | | | |
| --- | --- | --- | --- |
|  | **PSQI** | **CAR** | **BDNF** |
| **Anxiety disorder** |  |  |  |
| With (n = 9) | μ = 14.44 ± 0.99 | μ = 322.51 ± 85.95 | μ = 8997.52 ± 1875.30 |
| Without (n = 9) | μ = 15.77 ± 0.99 | μ = 420.27 ± 85.95 | μ = 14564.10 ± 1875.30 |
| **Personality disorder** |  |  |  |
| With (n = 13) | μ = 15.46 ± 0.86 | μ = 338.20 ± 72.53 | μ = 13143.55 ± 16.92.70 |
| Without (n = 4) | μ = 14.25 ± 1.55 | μ = 419.36 ± 130.77 | μ = 8132.6 ± 3051.56 |
