# Supplementary material for: Changes in Cortisol but Not in Brain-Derived Neurotrophic Factor Modulate the Association Between Sleep Disturbances and Major Depression
Source: Front Behav Neurosci. 2020 Apr 28;14:44. doi: 10.3389/fnbeh.2020.00044 (PMC7199815; doi:10.3389/fnbeh.2020.00044)
Supplement: Supplementary file 4 [file Table_4.docx]

Supplementary table 4- Spearman correlation test between molecular biomarkers and components of Pittsburgh sleep quality index (PSQI).

|  | **C1** | **C4** | **C5** | **C6** | **C7** |
| --- | --- | --- | --- | --- | --- |
| **CAR** | **-0.307*** | **-0.397*** | **-0.281*** | **-0.375*** | -0.24 |
| **BDNF** | 0.05 | 0.02 | 0.03 | 0.07 | -0.01 |

^BDNF: brain derived neurotrophic factor, CAR: Salivary cortisol awakening response, C1: subjective sleep quality, C4: sleep efficiency, C5: sleep alterations, C6: medication use, C7: daytime sleep dysfunction.^
