# Supplementary figures and images for: Changes in Cortisol but Not in Brain-Derived Neurotrophic Factor Modulate the Association Between Sleep Disturbances and Major Depression
Source: Front Behav Neurosci. 2020 Apr 28;14:44. doi: 10.3389/fnbeh.2020.00044 (PMC7199815; doi:10.3389/fnbeh.2020.00044)

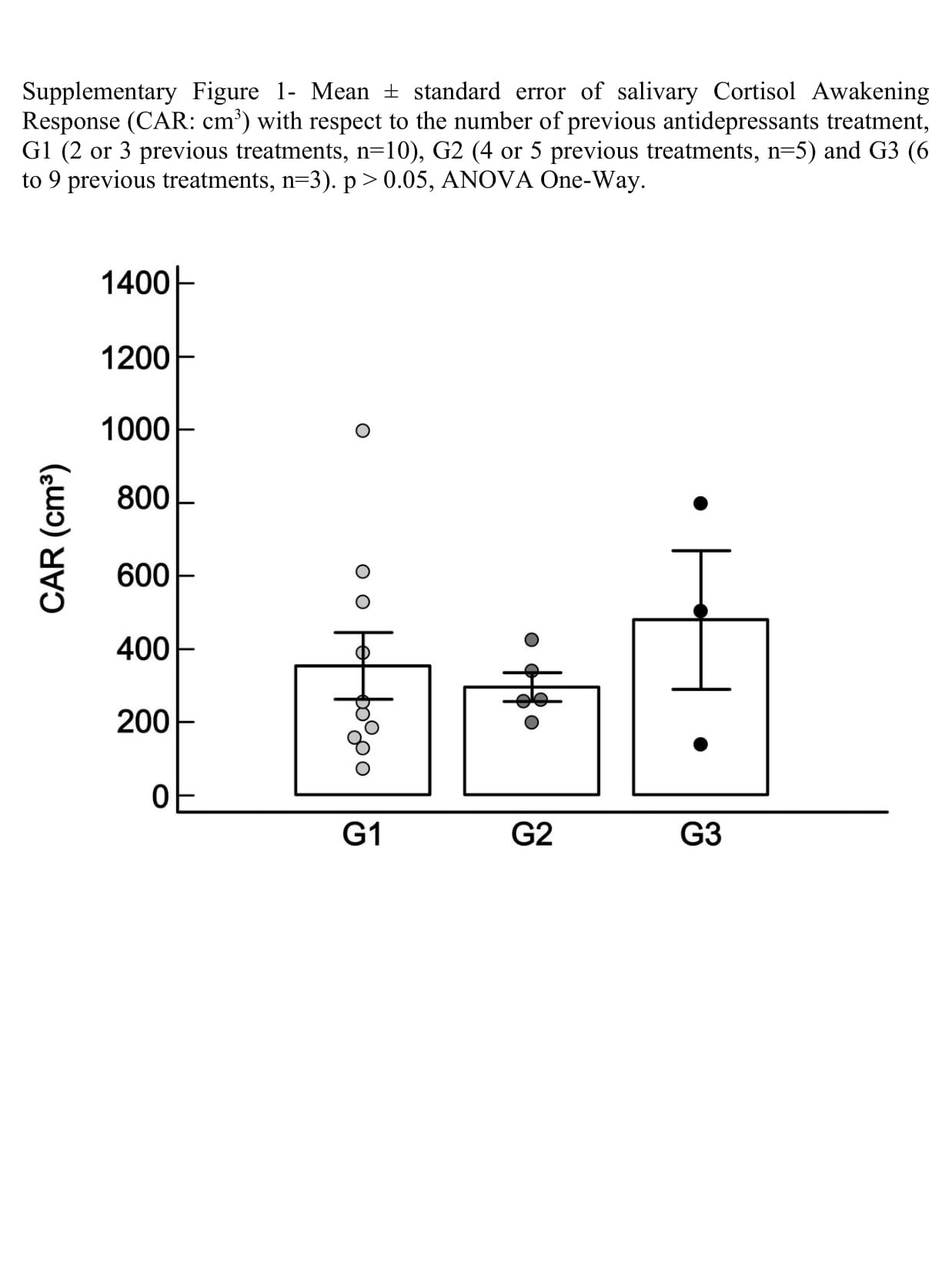

Supplement: Supplementary file 5 [file Image_1.JPEG]

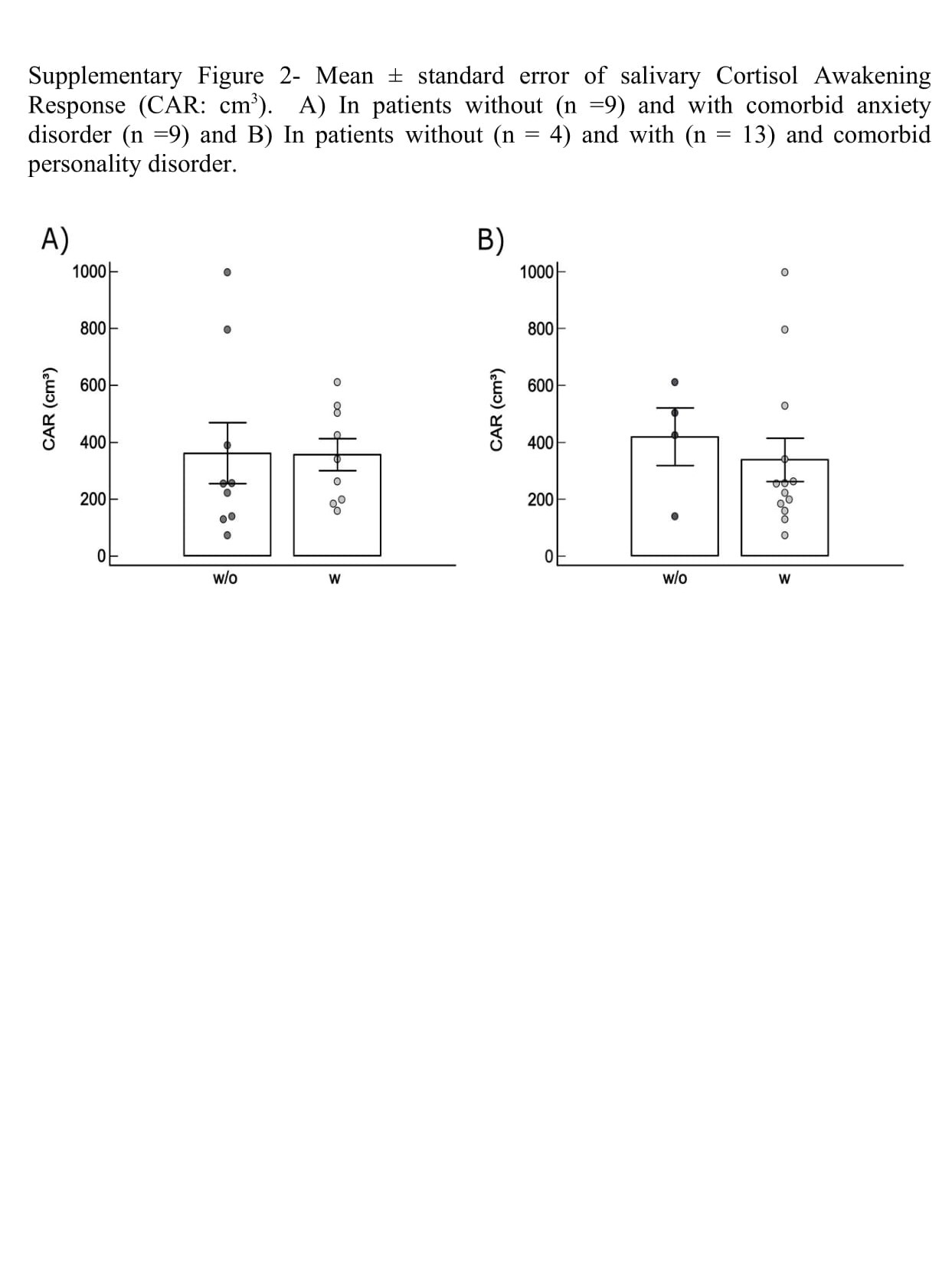

Supplement: Supplementary file 6 [file Image_2.JPEG]

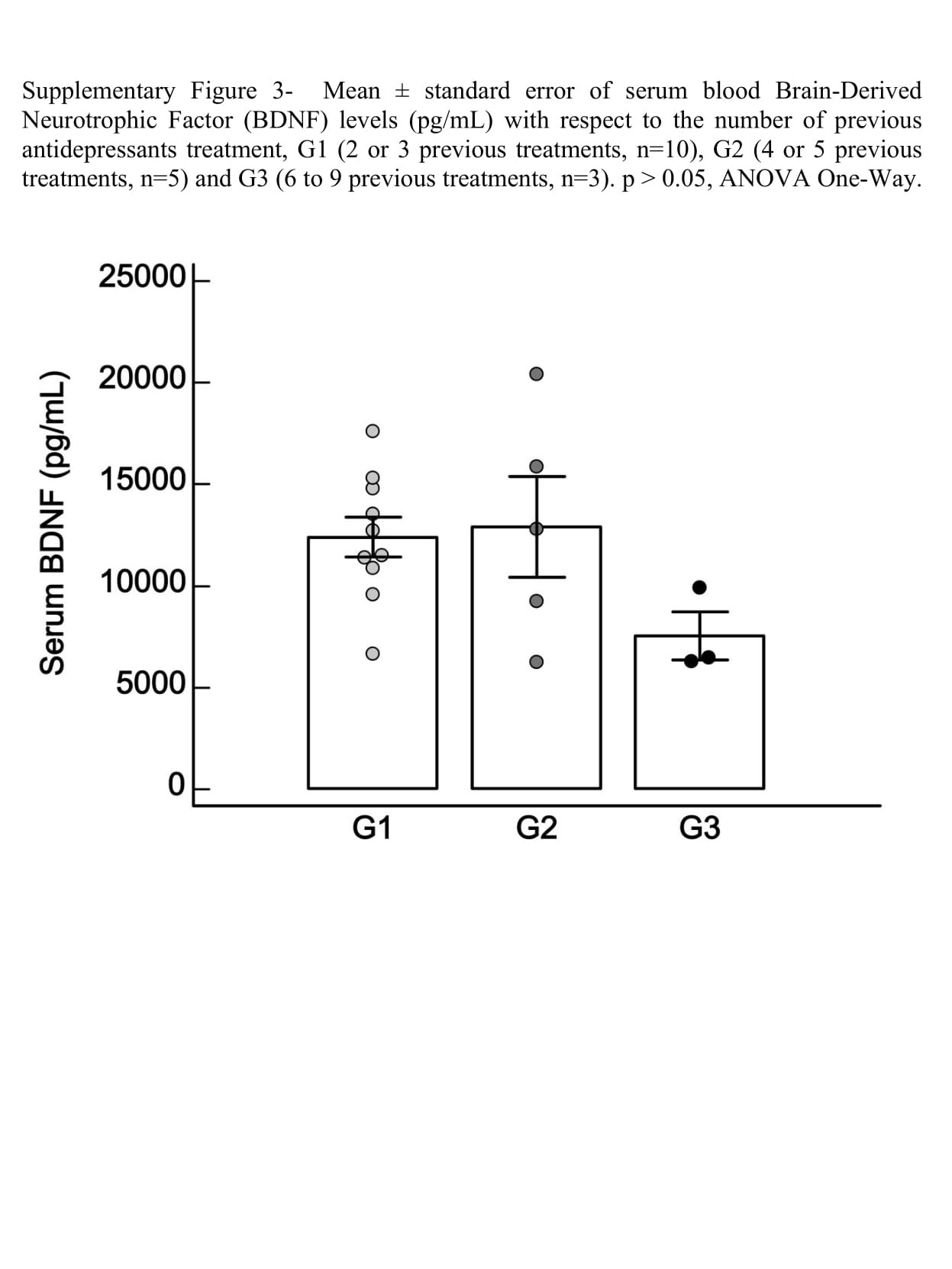

Supplement: Supplementary file 7 [file Image_3.JPEG]

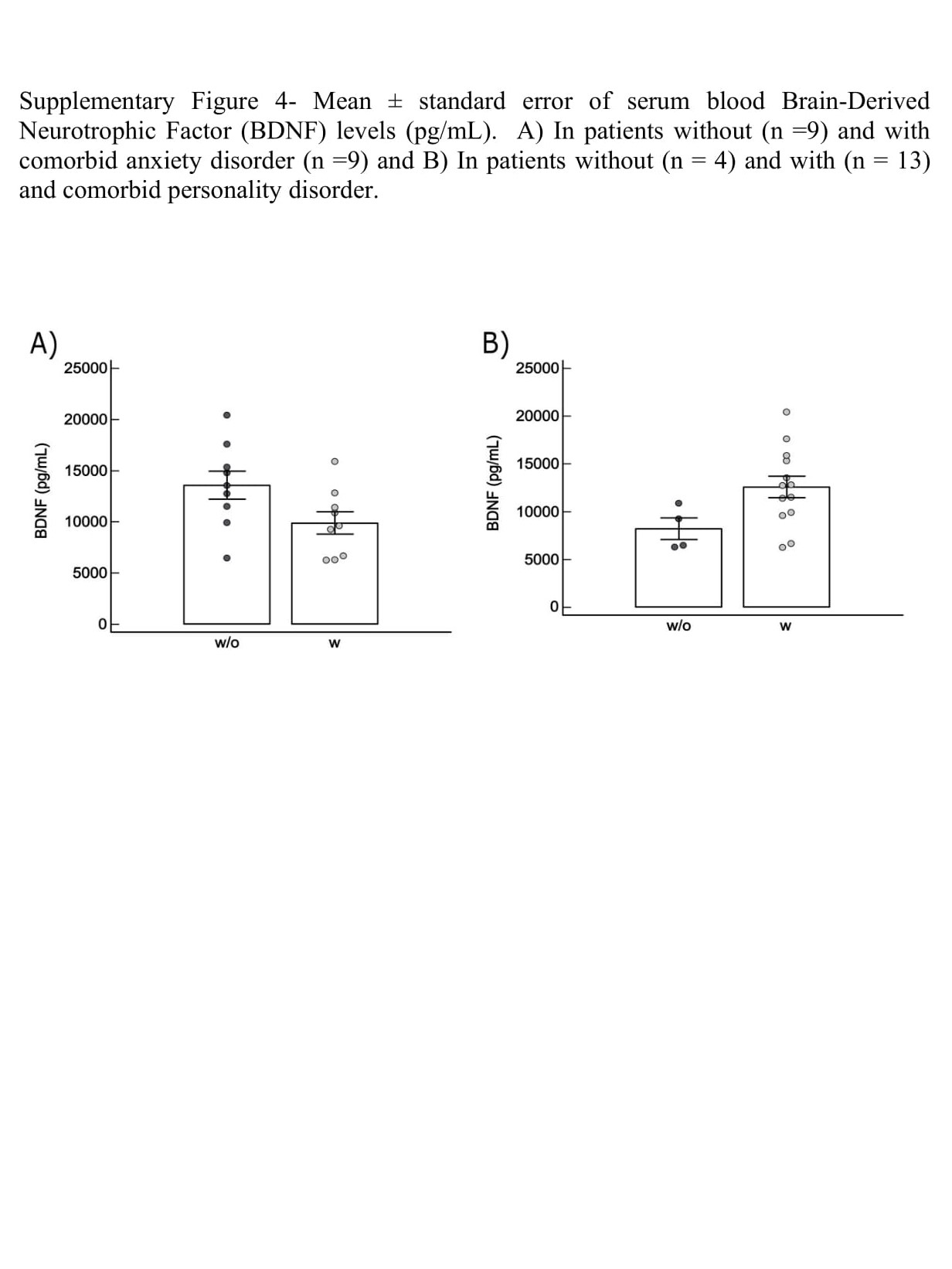

Supplement: Supplementary file 8 [file Image_4.JPEG]
